# Monthly Summary

**Published:** 1888-09

**Authors:** 


### Monthly Summary.

---

ERYTHROPHLÆINE AS A LOCAL ANÆSTHETIC.—Dr. Carl Koller has made some experiments with this substance, and finds that for some minutes after its application it produces pain, injection of the conjunctiva. When this has passed away the conjunctiva is quite insensible to touch or pin pricks, which lasts for several hours. This is followed by a cloudiness of the cornea, which continues for several days.—*Physician and Surgeon.*

---

EPISTAXIS: LEMON JUICE.—M. Geneuil (*Rev. sanidad militar.*), Madrid, for the past twelve years has employed injections into the nares of freshly-expressed lemon juice in rebellious cases of epistaxis depending upon disease of the liver and heart, and has never known it to fail in checking the hemorrhage, even after all ordinary means were ineffectual. He recommends it especially in children, who are much more difficult to tampon than adults. He first by means of a small urethral syringe, washes the nostrils free from coagula with water, and then injects freshly-prepared lemon juice. He rarely has to use more than one injection; but should the epistaxis not have ceased in two minutes, he repeats it. These results are not obtainable with a concentrated solution of citric acid. He considers this method as efficient, or even more so, than that of Verneuil's, which consists in bleeding from the hepatic region and it has over the latter the advantage that it is less difficult of application.—*Polyclinic.*

---

TREATMENT OF BURNS.—Nikolsky claims to have had excellent success in the treatment of burns of the second degree with fomentations of the following:

Tannin.
Alcohol (95) aa 4 grammes.
Rectified sulphuric ether 30 grammes.

The dressings are repeated two or three times a day. After the ether evaporates there remains a fine pellicle of tannin that causes prompt sedation of the pain and inflammation. The cure takes place much more rapidly than under the employment of collodion and other remedies in common use. The first dressing should be preceded by an antiseptic washing of the affected parts. The bullæ are punctured to allow the serum to escape, and the denuded surfaces are then powdered over with iodoform in a thin layer, and then dressed with the ether tannin.—*Journal de Medicine de Paris.*

